# Reliability and Validity of Kansas City Cardiomyopathy Questionnaire in Arabic Patients with Chronic Heart Failure

**DOI:** 10.3390/medicina59111910

**Published:** 2023-10-28

**Authors:** Ali M. Albarrati, Rayan Altimani, Osama Almogbel, Ali H. Alnahdi, Muneera M. Almurdi, Aliah Abuammah, Rakan Nazer

**Affiliations:** 1 Rehabilitation Sciences Department, College of Applied Medical Sciences, King Saud University, Riyadh 11451, Saudi Arabia; alialnahdi@ksu.edu.sa (A.H.A.); malmurdi@ksu.edu.sa (M.M.A.); 2 The Kingdom Hospital, Riyadh 11564, Saudi Arabia; rayanaltaimani@gmail.com; 3 Cardiac Sciences Department, College of Medicine, King Saud University, Riyadh 11461, Saudi Arabia; oalmogbel@ksu.edu.sa (O.A.); aabuammah@ksu.edu.sa (A.A.); raknazer@ksu.edu.sa (R.N.)

**Keywords:** quality of life, CHF, KCCQ, outcome measure, psychometric properties

## Abstract

*Background*: The Kansas City Cardiomyopathy Questionnaire (KCCQ) is the most specific and widely used questionnaire for assessing health-related quality of life (HRQoL) in chronic heart failure (CHF). This study aimed to examine reliability and validity of the KCCQ in Arabic patients with CHF. *Material and Methods*: Patients with CHF filled out the Arabic versions of the Minnesota Living with Heart Failure (MLHF) and KCCQ questionnaire, and performed a six-minute walk test (6MWT) on their first visit. On the return, the patients filled out the KCCQ along with the global rating of change (GRC) scale. Internal consistency, test–retest reliability, and construct validity were examined. *Results*: A total of 101 Arabic patients with CHF, with a mean (SD) age of 55 (11) years old, completed the study. The Cronbach’s alpha was 0.97, and the ICC_2,1_ = 0.95 (95%CI: 0.92 to 0.97, *p* < 0.001). The Arabic version of KCCQ was correlated with the MLHF (r = −0.57, *p* = 0.01) and with the 6MWT (r = 0.70, *p* < 0.001). *Conclusions*: The Arabic version of KCCQ is a reliable and valid measure of HRQoL, which could be utilized in routine clinical practice for Arabic-speaking patients with CHF.

## 1. Introduction

Chronic heart failure (CHF) is frequently associated with physical, functional, and mental impairments that can lead to a reduced quality of life [1]. Patients with CHF typically experience a lower health-related quality of life (HRQoL) compared to healthy individuals and those with other chronic diseases [2]. Heart failure clinicians typically use various physical, functional, and clinical indicators to evaluate and monitor the health status of their patients [3]. However, perceptions of health status in patients with CHF cannot be captured by different physical and clinical indicators alone, and may not be easily recognized by clinicians, which may warrant further assessment [4]. Therefore, it is extremely important to utilize patient-reported HRQoL measures in clinical practice to gain a comprehensive understanding of the health status of patients with CHF [3,4].

Improving the health status of patients with CHF, including their symptoms, function, and quality of life, is now widely recognized as a crucial primary outcome in the management of CHF [5]. Manifestations of CHF such as breathlessness, tiredness and fatigue contribute to a reduction in daily activities, exercise tolerance, and overall psychological well-being. These symptoms can lead to social isolation and decreased quality of life. The perception of quality of life varies among patients, and each patient may experience unique physical and psychosocial problems [4,5]. The methodology for quantifying HRQoL in CHF is evolving, and it has now advanced to shortened disease-specific scales.

A recent systematic review found that the Kansas City Cardiomyopathy Questionnaire (KCCQ) is the most specific and widely used self-reported questionnaire for assessing the quality of life in patients with CHF [6]. The KCCQ is a patient-reported outcome measure (PROM) that is specifically designed to evaluate the quality of care provided to patients with CHF [7]. The original KCCQ comprises 23 items, but a shorter version with 12 items has been developed for clinical purposes [7,8]. The KCCQ-12 includes domains that assess physical limitations, heart failure-specific symptoms (such as swelling, shortness of breath, and fatigue), quality of life, and the social impact of the disease [8]. These domains are used to generate two summary measures: the KCCQ-12 clinical summary score, which captures patients’ physical function and symptoms, and the KCCQ-12 overall summary score, which includes the physical, symptom, quality of life, and social function domains. The KCCQ has been used in recent clinical trials to assess the health status of patients with CHF, and to evaluate the effects of various pharmacological therapies and novel medical devices on the health status of patients.

In order to assess the extent to which patients are affected by the symptoms associated with CHF, healthcare professionals often use the KCCQ. This valuable resource allows healthcare providers to obtain a comprehensive picture of a patient’s level of functioning beyond merely measuring objective physical factors like reduced ejection fraction. Using tools like the KCCQ can greatly enhance clinicians’ ability to improve the quality of life of their patients with CHF.

The KCCQ-12 has been translated into several languages, including Arabic, and has been tested on patients with CHF from diverse ethnic backgrounds [9,10,11,12,13]. However, no study has yet examined the use of the KCCQ-12 in Arabic-speaking patients with CHF with reduced ejection fraction. Therefore, this study aimed to examine the reliability and validity of the Arabic version of KCCQ-12 among Arabic-speaking patients with CHF with reduced ejection fraction.

## 2. Materials and Methods

### 2.1. Study Design

This study was a psychometric property study.

#### 2.1.1. Setting and Participants

We recruited participants from outpatient cardiology clinics at King Saud University Medical City who were diagnosed with CHF with reduced ejection fraction, clinically stable, and were able to read or understand Arabic. In this study, we excluded participants with severe musculoskeletal, neurological, or respiratory diseases. All participants provided their consent and signed the consent forms. This study was approved by the ethical committee at King Saud University (IRB number: E-22-6642).

#### 2.1.2. Sample Size Estimation

We calculated the sample according to the consensus-based standards for the selection of health measurement instrument (COSMIN) guidelines [14]. The guidelines considered a sample size of 100 participants as an excellent number for examining the psychometric properties, including reliability and construct validity of the Arabic version of KCCQ-12.

#### 2.1.3. Physical and Clinical Measurements

Participants underwent anthropometric measurements, including height and weight, and their body mass index (BMI: kg/m^2^) was calculated. Vital signs were recorded for all participants using the Dinamap Carescape monitor V100 (GE HealthCare, Chicago, IL, USA) and their relevant clinical data were retrieved from their electronic medical records.

During the initial visit, all participants were requested to complete the Arabic version of KCCQ-12 and the Minnesota Living Heart Failure Questionnaire (MLHF), and performed a six-minute walk test (6MWT) as part of the initial assessment. Within 14 days from the initial visit, participants were asked to rate their overall health status since the last visit using a global rating of change (GRC) scale before completing the Arabic version of KCCQ-12 for a second time [15].

#### 2.1.4. The Kansas City Cardiomyopathy Questionnaire (KCCQ-12)

The KCCQ-12 is a 12-item self-reported questionnaire that measures physical function, symptoms, social function, and quality of life over the past two weeks [8]. The KCCQ-12 is a reliable and valid tool for measuring HRQoL in patients with CHF [8]. The 12-item KCCQ quantifies four domains of a patient’s health status related to heart failure: physical limitation (3 items), symptom frequency (4 items), quality of life (2 items), and social limitations (3 items). Item responses are coded in sequential order (1, 2, 3, etc.) from the worst to the best status. Each domain is scored and scaled from 0 to 100, with 0 indicating the worst possible health status and 100 the best. The health status is interpreted based on the total summary score of KCCQ as follows: very poor to poor (0–24), poor to fair (25–49), fair to good (50–74), good to excellent (75–100) [16]. The Arabic version of KCCQ-12 was obtained from the developer, and we obtained permission to use it.

#### 2.1.5. The Minnesota Living Heart Failure Questionnaire (MLHF)

The MLHF questionnaire comprises 21 questions that cover various aspects of how HF can adversely affect a patient’s life, including physical (8 items), emotional (5 items), and socioeconomic (8 items) [17]. Each question is scored on a scale of 0 to 5 to indicate the extent to which each specific adversity of heart failure has prevented the patient from living as they wanted to live during the past 4 weeks. The Arabic version of MLFH questionnaire is reliable and valid [18]. The MLHF is scored by summing up all 21 responses, with higher scores indicating a worse health status. The quality of life is classified based on a total score. A score of less than 24 indicates good, a score of 24–45 indicates moderate quality of life; and a score of more than 45 indicates poor quality of life [19].

#### 2.1.6. Six-Minute Walk Test (6MWT)

The 6MWT was conducted indoors, along a flat walkway corridor measuring 30 m, in accordance with the guidelines set by the American Thoracic Society [20]. Vital signs, including BP, HR, and SpO2 were measured before and after the 6MWT. The distance covered during the test was recorded in meters.

#### 2.1.7. Global Rating of Change Scale

The GRC is a reliable and valid tool for assessing changes in a patient’s health status over time, consisting of a single self-reported question [16]. In the present study, we scored the GRC on an 11-point scale that ranged from 5 to −5 points. A score of 5 indicates a significant improvement while a score of −5 indicates a significant worsening. The patient’s health status was considered stable if there was no change or an acceptable variability in the GRC scores ranging from 1 to −1 point.

### 2.2. Data Analysis

We conducted our analysis using SPSS Statistics for Windows, Version 26.0 (IBM Corp., Armonk, NY, USA). Continuous data are presented as means and standard deviations (SD), while categorical data are presented as frequencies and percentages. A statistically significant level was achieved with a *p* < 0.05.

#### 2.2.1. Floor and Ceiling Effects

Floor and ceiling effects of the Arabic version of KCCQ-12 were determined by calculating the percentage of participants who scored the lowest and highest, respectively. The floor effect was evident if more than 15% of the participants scored the lowest possible score, while the ceiling effect was established when more than 15% of the participants scored the highest possible score [21].

#### 2.2.2. Reliability

The Arabic version of KCCQ-12′s internal consistency and the test–retest reliability were measured using Cronbach’s α and the intraclass correlation coefficient (ICC_2,1_), respectively [21,22]. The standard error of measurement (SEM) for the Arabic version of KCCQ-12 was determined using the following equation: *SD*$\sqrt{1-\mathrm{ICC}}$, which was then used to calculate the minimum detectable change with a 95% confidence interval (MDC_95_) using the equation: *MDC*_95_ = 1.96 × $\sqrt{2}$ × *SEM*. In addition, we used the Bland–Altman analysis to assess the level of agreement between the two measurements of the Arabic version of KCCQ-12 on both visits [23].

#### 2.2.3. Construct Validity

The Arabic version of KCCQ-12′s construct validity was measured by comparing it with the MLHF and the 6MWT. We hypothesized that the Arabic version of KCCQ-12 would have a moderate negative association (r > 0.40) with the MLHF, and a moderate positive association (r > 0.40) with the 6MWT. Pearson’s correlation coefficient was used to measure the strength of the associations between these variables.

## 3. Results

### 3.1. Physical and Clinical Characteristics of Participants

On the initial visit, a total of 101 participants (81 males) filled out the Arabic versions of KCCQ-12 and MLHF, and also underwent the 6MWT. Participants’ physical and clinical characteristics are demonstrated in Table 1. Following an average interval period of 12 days from their initial visit, participants completed the KCCQ-12 for a second time. Prior to this, they answered the GRC scale to assess their clinical stability.

### 3.2. Floor and Ceiling Effects

Out of 101 participants, 10 participants scored the highest on the Arabic version of KCCQ-12, while only 3 participants scored the lowest. Therefore, there was no evidence of floor or ceiling effect issues associated with the Arabic version of KCCQ-12.

### 3.3. Reliability

The Arabic version of KCCQ-12 revealed excellent internal consistency with a Cronbach’s α of 0.97 and test–retest reliability with an ICC_2,1_ of 0.95 (95%CI: 0.92 to 0.97, *p* < 0.001). The SEM was 1.5 points, and the MDC_95_ was 4.3 points.

The Bland–Altman analysis showed a mean difference between the two measurements of −0.9 (95%CI: −2.67–0.81) with an SD of 6.8. The correlation between the two measurements was r = 0.955, *p* < 0.001. The limits of agreement were between −12.86 and +11.86. The analysis showed that there was no bias between the measurements with only minimum random errors.

### 3.4. Construct Validity

The Arabic version of KCCQ-12 was associated with the MLHF (r = −0.57, *p* = 0.01). The KCCQ-12 physical domain was associated with the 6MWT (0.42, *p* < 0.001). Both HRQoL outcome measures, KCCQ-12 and MLFH, were associated the 6MWT, r = 0.70 and r = −0.60, all *p* < 0.001, respectively.

## 4. Discussion

This study explored the reliability and validity of the Arabic version of KCCQ-12 in Arabic-speaking patients with CHF. The Arabic version of KCCQ-12 had excellent reliability, including internal consistency and test–retest reliability, and it had good validity. Additionally, the Arabic version of KCCQ-12 revealed to be well understood with quick completion.

CHF is prevalent in the Middle East with an estimated prevalence rate of 2% [24]. However, the incidence rate is 10 years younger, at a mean age of 57 years, compared to their counterparts in the developed countries [24]. Additionally, it is a common cause of hospital admissions, and is associated with a significant reduction in quality of life and increased healthcare costs. Assessing symptoms along with general health status is critical to the management of CHF. The need for easy-to-use, reliable, and validated assessment tools led to the development of a disease-specific instrument that evaluates the patients’ subjective perspective on CHF, the KCCQ-12.

The Arabic version of KCCQ-12 had similar reliability to the original version of KCCQ-12. Spertus and Phillip [8] measured the reliability of KCCQ-12 on 79 stable patients with CHF within 6-week intervals between the measurements and found high reliability for the KCCQ-12 summary score with an ICC of 0.92. The MDC for the Arabic version of KCCQ-12 is similar to the score reported by the developer of the original questionnaire at 4.7 points. A minimum clinically important difference of ≈−3 to 5 points for the original KCCQ-12 summary score is considered a cut-off value for deterioration and improvement, respectively.

The KCCQ-12 has been approved as an outcome measure for CHF surveillance and prognostication [16]. Measurement of HRQoL in integration with the clinical examination provides clinicians with information about a patient’s health status and how the disease impacts it [25]. In addition, it guides clinicians to monitor the impact of the treatment on the patient’s HRQoL and whether the patient has improved or deteriorated since the last visit [26,27,28].

In the current study, we used the most common disease-specific HRQoL scale in the literature, the MLHF, to evaluate the construct validity of the Arabic version of KCCQ-12 [6]. The Arabic version of KCCQ-12 showed a moderate inverse correlation with the MLHF. This indicates that patients with CHF with high scores on the KCCQ-12 would score low on the MLHF, reflecting better HRQoL. The MLHF is a disease-specific HRQoL specially designed for patients with CHF and covers similar quality of life aspects to the KCCQ-12, including clinical symptoms, physical function, and psychosocial. A recent study reviewed all HRQoL outcome measures in CHF and found that the KCCQ and MLHF are the most specific and widely incorporated in clinical research [6]. Previous validation studies have compared the original KCCQ-23 with other HRQoL outcome measures, including the Short-Form-36, the MLHF, the EuroQol-5D, and the New York Heart Association (NYHA) classification [9,10,11,12,13]. However, there is no study so far that has compared the KCCQ-12 with other quality of life scales in patients with CHF, which makes the comparison impossible.

Managing CHF requires a multifaceted approach aimed at improving quality of life while minimizing disability and mortality [26]. Assessment of the patient’s functional status is essential in determining the severity of disease and for guiding treatment decisions. In clinical practice, the KCCQ and 6MWT are commonly used assessments for patients with CHF. These evaluations not only provide valuable information about a patient’s physical functionality, but also their quality of life. In our study, we found over a third of the patients had poor health status, which influenced their functional capacity, though the patients were stable and had no change in their medications. Incorporating these clinical tools with the initial diagnosis of heart failure provides a deeper understanding of the patient’s situation to the clinicians, helping them make informed decisions, and saves hospital costs, especially with the era of tele-health [27].

With the rapid transformation toward tele-health, integrating the KCCQ in digital healthcare systems for patients with CHF would provide clinicians with an insight into the health status of patients who would not be captured by other physical and clinical tests [5,28]. Given the prognostic value of the KCCQ scores for hospitalization and mortality, the changes in KCCQ scores over time direct the attention of healthcare providers toward optimizing the health status of their patients [29,30]. A study by Pokharel and colleagues [25] found that the recent score of the KCCQ was the most prognostic than previous scores and was associated with subsequent clinical events. A number of longitudinal studies have reported an inverse relationship between health status as measured by the KCCQ and cardiac biomarkers such as N-terminal pro-B-type natriuretic peptide (NT-proBNP) in patients with CHF with reduced ejection fraction [31,32,33]. NT-proBNP is considered a key test in the diagnosis, intervention, and prognosis of CHF as it reflects cardiac structure remodeling and provides a valuable clinical picture about cardiac structure changes and effectiveness of medications over time on follow-up visits. Interventional studies reported that reduced NT-proBNP levels were associated with an improvement in health status in patients with CHF with reduced ejection fraction [31,32].

The healthcare provided to patients with CHF varies markedly across clinicians and some patients with CHF do not receive the recommended care [34,35]. Therefore, implementing the KCCQ in tele-health systems would identify patients who need a referral to more advanced specialists for further management and reduce the risk of clinical events [5,26,27]. Heart failure nurse specialists play an integral part in the management of patients with CHF in outpatient settings, and could utilize this simple health status tool to monitor the patient’s health status and determine if the patient needs to be seen by another healthcare provider or consult with a heart failure physician to optimize medical treatment.

This study should acknowledge a number of limitations. The majority of our sample was male but nearly a quarter of the sample was female. In this study, we only recruited stable patients with CHF with reduced ejection fraction. In addition, the responsiveness of the Arabic version of KCCQ was not examined in the current study, and therefore it would be necessary to assess its responsiveness in future research.

## 5. Conclusions

The Arabic version of KCCQ-12 is a well-understood scale with high test–retest reliability and good validity in Arabic-speaking patients with CHF. The Arabic version of KCCQ-12 is suitable for all Arabic-speaking patients with CHF, and has the potential to be implemented in daily practice, guide clinicians to provide tailored care, and optimize the management of patients with CHF.

## Figures and Tables

**Table 1:** Physical and clinical characteristics of patients.

	Mean	SD
Age (years)	55	11
BMI (kg/m^2^)	31.36	4.46
Marital status		
Single	13 (13%)	
Married	83 (82%)	
Divorced	5 (5%)	
Education		
Elementary	5 (4%)	
High school	60 (59%)	
Bachelor	33(32%)	
High Education	6 (5%)	
KCCQ 12 Domains		
KCCQ Physical	63.33	28.76
KCCQ Symptom	83.97	23.07
KCCQ Quality of life	79.05	27.39
KCCQ Social	73.68	32.01
KCCQ Clinical summary score	77.50	22.82
KCCQ-12 Overall summary score	76.93	22.27
KCCQ-12 (0–24)	3 (3%)	
KCCQ-12 (25–49)	15 (15%)	
KCCQ-12 (50–74)	23 (23%)	
KCCQ-12 (75–100)	60 (59%)	
MLHF	17.48	8.67
MLHF (0–24)	73 (72%)	
MLHF (25–45)	16 (16%)	
MLHF (>45)	12 (12%)	
6MWT (m)	315	90

Abbreviations: BMI: body mass index; KCCQ: Kansas City Cardiomyopathy Questionnaire; MLHF: Minnesota Living Heart Failure Questionnaire; 6MWT: six-minute walk test.

## Data Availability

The data supporting the findings of this study are available from the corresponding author, A.M.A., upon reasonable request.
